# Microsatellite instability assessment is instrumental for Predictive, Preventive and Personalised Medicine: status quo and outlook

**DOI:** 10.1007/s13167-023-00312-w

**Published:** 2023-01-25

**Authors:** Jakub Styk, Zuzana Pös, Ondrej Pös, Jan Radvanszky, Evelina Hrckova Turnova, Gergely Buglyó, Daniela Klimova, Jaroslav Budis, Vanda Repiska, Bálint Nagy, Tomas Szemes

**Affiliations:** 1 Institute of Medical Biology, Genetics and Clinical Genetics, Faculty of Medicine, Comenius University, 811 08 Bratislava, Slovakia; 2 Comenius University Science Park, 841 04 Bratislava, Slovakia; 3 Geneton Ltd, 841 04 Bratislava, Slovakia; 4 Institute of Clinical and Translational Research, Biomedical Research Centre, Slovak Academy of Sciences, 845 05 Bratislava, Slovakia; 5 Department of Molecular Biology, Faculty of Natural Sciences, Comenius University, 841 04 Bratislava, Slovakia; 6 Slovgen Ltd, 841 04 Bratislava, Slovakia; 7 Department of Human Genetics, Faculty of Medicine, University of Debrecen, 4032 Debrecen, Hungary; 8 Slovak Centre of Scientific and Technical Information, 811 04 Bratislava, Slovakia; 9 Medirex Group Academy, NPO, 949 05 Nitra, Slovakia

**Keywords:** Microsatellite instability, Cancer, Screening, Massively parallel sequencing, Liquid biopsy, Patient stratification, Predictive Preventive Personalised Medicine (PPPM / 3PM)

## Abstract

A form of genomic alteration called microsatellite instability (MSI) occurs in a class of tandem repeats (TRs) called microsatellites (MSs) or short tandem repeats (STRs) due to the failure of a post-replicative DNA mismatch repair (MMR) system. Traditionally, the strategies for determining MSI events have been low-throughput procedures that typically require assessment of tumours as well as healthy samples. On the other hand, recent large-scale pan-tumour studies have consistently highlighted the potential of massively parallel sequencing (MPS) on the MSI scale. As a result of recent innovations, minimally invasive methods show a high potential to be integrated into the clinical routine and delivery of adapted medical care to all patients. Along with advances in sequencing technologies and their ever-increasing cost-effectiveness, they may bring about a new era of Predictive, Preventive and Personalised Medicine (3PM). In this paper, we offered a comprehensive analysis of high-throughput strategies and computational tools for the calling and assessment of MSI events, including whole-genome, whole-exome and targeted sequencing approaches. We also discussed in detail the detection of MSI status by current MPS blood-based methods and we hypothesised how they may contribute to the shift from conventional medicine to predictive diagnosis, targeted prevention and personalised medical services. Increasing the efficacy of patient stratification based on MSI status is crucial for tailored decision-making. Contextually, this paper highlights drawbacks both at the technical level and those embedded deeper in cellular/molecular processes and future applications in routine clinical testing.

## Introduction

Microsatellite instability (MSI) is defined as alterations in the length of short tandem repeats (STRs) or microsatellites (MSs) found across the whole genome. Simple, canonical MSs comprise repetitions of a short sequence (usually defined as 1 to 6 nucleotides) that were associated with human cancer in the 1990s for the first time [1–3], compound MSs contain two or more different repeat units, while complex motifs display varied lengths of repeat units and may include interruptions [4].

MSs have long been viewed as an important aspect of human genetic variation that may contribute to the personalised management of many diseases including intellectual disability, cancer, diabetes and cardiovascular diseases [5, 6]. Accurate assessment of the role of MSs in human conditions displaying genetic heterogeneity is difficult due to the poorly defined copy numbers in human reference genomes [7].

In certain types of cancer and associated conditions, MSs display genome-wide instability, i.e. they tend to accumulate indels of several bases due to a failure of the DNA mismatch repair (MMR) machinery. MSI is detected in about 10–15% of colorectal (CRC), gastric and endometrial cancers (EC), while found less frequently in other solid tumours [8, 9]. This replication error phenotype is considered to be a hallmark of a hereditary cancer-prone syndrome, increasing individual risk for various malignancies. It is called Lynch syndrome (LS) [10] and MSI occurs in approximately 90% of cases [11, 12]. Tumours are conventionally classified according to the proportion of unstable STR markers into the following classes: (i) MSI-H (microsatellite instability-high), (ii) MSI-L (MSI-low) and (iii) MSS (microsatellite stable) [13]. Redford et al. [14] suggested replacing the above 3 classes by a binary assessment of MSI events. This would fit-in well with the findings that MSI-L CRCs are often clinically similar to MSS CRCs [15]. Furthermore, patients with MSI tumours show an upregulated expression of various immune checkpoints, currently under study as potential therapeutic targets. They provide better prognosis in the early stages of CRC and a limited benefit from 5-fluorouracil adjuvant chemotherapy in stages II and III [16]. We believe the core of personalised treatment depends on identifying cases where clinicians can effectively utilise the targeted therapeutic procedures and agents to increase the patient’s lifetime follow-up. That is the way how the diagnosis and management of individuals with LS or their at-risk relatives should move from reactive testing to proactive Predictive, Preventive and Personalised Medicine (3PM). This shift is fundamental to improve patient outcomes and deliver socio-economic benefit [17, 18].

MSI detection methods evolve continuously, the mechanisms and relationship between MSI and tumours are better understood, and more possibilities for their clinical application are emerging. Earlier methods to determine MSI status, such as immunohistochemistry (IHC) and MSI-PCR [3, 19–22], are still widely used. Apart from certain technical limitations, the full informational potential of MSI status is limited mainly by the need for both tumour and healthy samples for analysis. As such, they are applicable when characterising known and surgically accessible tumours. Non-invasive techniques, however, have started to complement conventional and routinely used invasive methods, exploiting the vast potential of highly sensitive massively parallel sequencing (MPS) coupled with liquid biopsy sampling. Their main selling point is the future possibility of analysing tumours with no need of a priori awareness of their existence regardless of surgical accessibility or the availability of matching normal tissue for parallel analysis. These will enhance both screening and diagnosis in oncogenetics, with a tremendous impact of 3PM across various levels of healthcare [23, 24].

Acknowledging the limitations we have to face in a rapidly growing field, in this review, we would like to provide an up-to-date description of MSI detection strategies and computational tools which allow the processing of high-throughput MPS data for MSI screening and characterisation. According to fact-based evidence, we hypothesise how the routine MSI diagnosis could be advanced to a new age of novel 3PM-based procedures.

## The origin of MSI events in cancer

Human genome is a highly dynamic structure, constantly accumulating DNA damage which leads to mutation. One of the essential mechanisms to ensure an acceptable level of whole-genome integrity is a robust and evolutionarily fine-tuned error detection and elimination mechanism called mismatch repair system (MMR). It consists of several proteins encoded by MMR genes. The four most well-known include *MLH1* (mutL homologue 1), *MSH2* (mutS homologue 2), *MSH6* (mutS homologue 6) and *PMS2* (PMS1 homologue 2) [25, 26].

Due to a relatively high complexity of this post-replication system, inter-individual and tumour-specific variability in MMR efficiency is considerable [27]. Loss-of-function mutations in MMR genes may lead to an impaired MMR system, i.e. MMR deficiency (dMMR), which may result in unique somatic alteration events in the length of MS loci, known as MSI [28]. In normal cells, the MMR verifies and maintains the repeat count of MSs. The results of its failure are seen in MSI-positive tissues: an accumulation of mutations that may affect the function of many oncogenes and tumour suppressor genes [29, 30]. This, in turn, is followed by a genome-wide accumulation of frameshift and missense mutations resulting from insufficient correction of base–base mismatches and insertion/deletion (indel) loops that occur in cancer genomes as a result of replication errors or slippage events. MMR deficiency also affects MS sequences, which represent, as pointed out above, highly unstable genomic regions [31].

It has been estimated that replicative polymerases make an error on average once every 10^4^–10^5^ nucleotides. Thus, in every single mammalian cell genome, approximately 100,000 replication errors occur during each cell division. Due to their 3′–5′ exonuclease activity, human DNA polymerases, Pol δ and ε, keep, check and reduce this error rate to 1 in 10^5^–10^7^ bases. However, the MMR system may further reduce replication mismatches to about 1 in 10^9^–10^10^ synthesised nucleotides [32–34]. Surprisingly, MMR is more proficient in removing replication errors on the lagging strand [35, 36].

The MMR complex itself is composed of two types of heterodimers. The first one is MutS, built from MSH2/MSH6 or MSH2/MSH3 pairs, resulting in MutSα and MutSβ complexes, respectively. The MutSα complex recognises mismatches and indels of 1–2 bp, while MutLβ is able to identify indels of larger sizes [37]. Initiation of the repair mechanism by MutSα and MutLβ is followed by the recruitment of MutL, the second heterodimer, composed of MLH1 in a complex with PMS2, PMS1 or MSH3, giving rise to the MutLα, MutLβ or MutLγ complexes, respectively, which catalyse the excision of mismatches and error-free resynthesis of DNA [38, 39].

## Chromatin organisation affects the frequency of MSI

It should be noted that MSI restricted to single loci or even to single alleles may result from the length of individual MS motifs themselves. This happens when MS repeats exceed certain stability thresholds (MMR deficiency is not required). Such single allele MSIs are typical for repeat expansion disorders, in which the instability of a locus may gradually increase with a growing number of repeats. Typically, one large unstable motif develops (premutation or a fully expanded allele) while other motifs of the same genome, or even the second allele of the same locus, remain stable [40–42]. In contrast, conventional MMR-deficiency-dependent MSI exerts genome-wide effects on MSs, although this effect seems highly uneven. It has been observed that the local density of MSI events is inversely correlated with that of mutations overrepresented in actively transcribed regions. The relative depletion of MSI at stable nucleosome positions also supports the notion that chromatin configuration is a major determinant of the genomic distribution of MSI, at least in CRCs and a subset of uterine corpus endometrial carcinomas [43]. A follow-up study evaluating whole genomes, most of them stated as MSI-H, revealed a high ratio of MSI events in DNA regulatory elements like promoters and enhancers, in actively transcribed areas (euchromatic regions), and within intergenic, intronic and 3′ untranslated regions (UTR), contrary to non-transcribed constitutive heterochromatin [44].

Nevertheless, there is considerable inconsistency in the definition of which genome regions are more or less prone to presence of MSI events. The interpretation of affected domains largely depends on the approach used. Further research is needed to understand whether the increased MSI frequency in open chromatin-like domains occurs during or after DNA replication. There are, however, some possible explanations for the increased MSI occurrence in open chromatin domains: (i) the proofreading activity of DNA polymerase may depend on the state of chromatin; (ii) replication in these domains may proceed more quickly, resulting in a higher error rate; (iii) chromatin-dependent DNA polymerase fidelity may significantly reduce the number of MSI events in nucleosome-occupied regions; and (iv) the base composition of the sequence around an MS site also significantly impacts the susceptibility of the repeat to expansion [45].

According to some evidence, MS’ impact on chromatin state is frequently mediated by regulatory changes of histones. Histone tags that open up chromatin and increase transcriptional activity, such as H3K4me3, H3K36me3 and H3K9ac, are associated with an increased prevalence of MSI events, compared to H3K9me2, H3K9me3 and H3K27me2, which act as transcriptional repressors [43]. A genome-wide analysis of STRs affecting gene expression demonstrated that they were strongly associated with histone modifications responsible for transcriptional activation — H3K4me3, H3K27ac, H3K36me3 and H3K9ac. Conversely, they were found to be depleted near the H3K27me3 mark linked to transcriptional repression [46].

Hause et al. [47] published data showing that in certain tumours, MSI accumulates in some functional areas of the genome, such as ion-binding genes. The activin A receptor type 2A (*ACVR2A*) [48, 49] and ring finger protein 43 (*RNF43*) [50] with coding repeat sequence A(8) or C(7) were found to be frequently affected in MSI-positive tumours compared to MSS tumours. To sum it up briefly, studies suggest relevant differences between the MSI-H and MSS groups, indicating the importance of MSI patterns across two levels (within and/or between tumour types). This suggests that they could be used to develop new pan-cancer panels with much greater detection capability. In addition, in 67–100% of evaluated samples classified as MSI-H, the homopolymer repeat located within the splicing region of the *ORC4* gene was also found to be unstable [47].

## Role of homopolymeric sequences in MSI

It has been almost two decades since researchers uncovered the vast potential of homopolymeric marker sites as the most interesting type of STRs. Despite the conserved nature of coding homopolymer repeats [51], the non-coding repeats may vary significantly among different individuals. However, if no polymorphisms are known and the number of recurring units at a locus is identical and constant in almost all individuals, even non-coding homopolymers could be seen as quasi-monomorphic. In CRC patients, three of the five well-known quasi-monomorphic monomeric tracts (BAT-25, BAT-26 and NR-21) are relevant in evaluating MSI events [52].

From all types of STR sequences, the most copious ones in the human genome are homopolymers, predominantly poly(A) and poly(T) tracts [53, 54]. A recent regression analysis proved that motifs composed of A/T have a higher error rate compared to G/C, making them the most mutated MS sequences in humans [55]. These results suggest that the association between A/T stretches and cancer may have developed due to the occurrence of MSI.

Indels in polypyrimidine tracts located in the 5′ immediate neighbourhood of a donor splice site may cause exon skipping [56, 57], whereas changes in the 5′ and 3′ UTR affect transcriptional efficiency and mRNA stability, respectively [58]. Evidence suggests that a poly(A) tract of 30 bp in the promoter of the *HGF* gene is associated with a compactly packed promoter in normal tissue and thus inaccessible to DNA cleaving enzymes. However, deletions in this poly(A) repeat loosen the chromatin structure, modify protein binding and transcriptionally stimulate the promoter in breast cancer tissue [59]. In this context, a comprehensive Selective Targets database, called Seltar*base*, was created to collect all known mutations in coding, non-coding, UTR and intronic homopolymeric tracts studied in MSI-positive tumours of different organs (http://www.seltarbase.org/). It enables identification of relevant genes for tumorigenesis based on their mutation frequency [60]. However, its relevance is declining since the database content has not been updated for almost 10 years.

## Clinical significance of MSI detection in cancer management and therapeutic decision-making

Detection of MSI events has broad clinical applications during the lifetime follow-up of CRC, enabling (i) a better management of MSI-positive tumours, and therefore, of LS patients and their relatives at risk; (ii) a better prognosis for patients with MSI-H CRC compared to those with MSS lesions; (iii) omission of fluorouracil, carboplatin or cisplatin adjuvant chemotherapy in cases exhibiting MSI (as they tend to respond poorly) [61, 62]; and (iv) a prediction for the efficacy of immune checkpoint inhibitors (ICIs) in order to identify patients in whom such personalised treatment may be beneficial [63]. The consequence of impaired efficacy of the MMR system is a tumour mutation burden (TMB), which may lead to a high neoantigen load. These are capable of triggering an anti-tumour immune response, which accounts for ICI sensitivity [64–66]. Nonetheless, in recent years, the development of personalised ICI therapies that target neoantigens has been drawing attention in the field of customised cancer vaccines [67]. This type of specific immunotherapy might deliver clinical benefits. Cost optimisation and time-to-production are as yet challenging; nevertheless, there is hope for laboursaving personalised treatment with a tremendous impact on the socio-economic burden in developing 3PM-based oncology.

In a study analysing MSI status in exomes, Hause et al. observed that the number of unstable MS loci in a tumour exome correlates with patient survival [68, 69]. Such distributions of global instability may be more informative than conventional approaches in clinical management of cancer patients and their treatment. Previous studies have shown that MSI-positive tumours are burdened with neo-antigens easily recognised by the immune system. The effectiveness of PD-1 inhibitors against solid MSI-H tumours exceeds its efficiency compared to MSI-L/MSS cases. In addition to inactivating the genes involved in tumour suppressor pathways as a consequence of dMMR, tumour cells produce neo-peptides known as immunogenic frameshift peptides caused by indels occurring in coding MSs [70, 71].

Abida et al. [72] showed that patients with MSI-H prostate cancer have a good prognosis with the anti-PD-1/PD-L1 treatment, similarly to MSI-H CRC patients. Among the various antibodies, pembrolizumab and nivolumab, two agents that target PD-1/PD-L1 and CTLA-4 immune checkpoint components, have been shown to be efficient across different types of cancer [73, 74]. Currently, a consensus concerning the benefit of such neo-adjuvant therapies seems to have been reached for patients with MSI-H tumours; however, there are still many open questions regarding treatment duration, the timing of surgery and the efficiency of anti-PD-1 therapy alone versus a combination with anti-CTLA-4 therapy [75]. Such clinical studies also prove that MSI testing is adequate in malignancies that are not routinely recommended for MSI screening. Surprisingly, almost 35% of all MSI-H tumours were found to be non-CRC [76] and these patients may benefit from early diagnosis as well as personalised medical services and 3PM.

Apart from the patients’ response to immunotherapy, it is also important to note that MSI-H tumours are enriched in altered signalling pathways, rendering the patient less likely to respond. Factors that may impair response include disrupted JAK/STAT signalling [77, 78] and activation of WNT signalling [79–81]. Studies focusing on such pathways may inform about combination therapies and identify not only patients who are likely to respond well to immunotherapy due to dMMR, but also patients who are equally likely to respond for a different reason: a high neoantigen burden unrelated to dMMR. This may help improve the stratification of patients and treatment regimens, thus delivering higher 3PM efficiency.

On a side note, the phenomenon of MSI was reported in diseases other than cancer, such as neurodegenerative disorders [82] (which are now recognised as repeat expansion disorders), atherosclerosis [83, 84] and in spontaneously aborted embryos [85]. In these disorders, however, MSI is not evaluated as part of the diagnostic process as it is in cancer.

## High-throughput strategies in MSI screening

Methods suitable for MSI detection range from conventional assays [8], such as capillary gel electrophoresis based on comparing the fragment profile of an amplified set of five quasi-monomorphic homopolymer loci (see Table 1) and IHC [86], through high-resolution melting analysis (HRM) [87, 88] and droplet digital PCR (ddPCR) to high-throughput sequencing platforms [89] (see Table 1 and Table 2). The power of MPS in research and routine clinical settings has been well established and extensively discussed in the literature, at least for SNVs (single nucleotide variants) and CNVs (copy number variants) [90, 91]. Compared to the above, the MPS-based detection and characterisation of TRs took longer to catch on, most likely due to high allelic variability in individual TR loci and certain initial technical limitations. Numerous other challenges also need to be overcome, including issues related to the PCR-induced stutter effect producing artefacts that appear as minor peaks of one or more repeat units, preferential amplification of shorter alleles, overall amplification of larger alleles and the existence of complex motifs. Following the first studies exploiting MPS data for TR characterisation, large-scale genome-wide TR detection began to spread across different biomedical areas, from monogenic repeat-expansion disorders [92, 93] to complex multifactorial traits and diseases [94, 95]. A few years ago, it was confirmed that tumour sequencing, as a first step in the diagnosis of cancer predisposition syndromes such as LS, could serve as an alternative screening assay even for patients with CRC [96].

**Table 1:** List of assays for the assessment of MSI

Assay	Methods/Lab. equip	Analysis	Markers (MNRs/DNRs)	Classification of MSI status	Sample/input	Paired samples	Ref.
MSI Analysis System, 1.2 (Promega)	FM-PCR, Capillary Electrophoresis/compatible Genetic Analyzer	Fragment Analysis Software (e.g. GeneMapper®)	BAT-25, BAT-26, NR-21, NR-24, MONO-27/PentaC, PentaD	Ternary: MSI-H (2 ≥ /5); MSI-L (1/5); MSS (0/5)	FFPE/1–2 ng DNA	R	[169]
OncoMate™ MSI Dx Analysis System (Promega)	FM-PCR, Capillary Electrophoresis/compatible Genetic Analyzers	Fragment Analysis Software	BAT-25, BAT-26, NR-21, NR-24, MONO-27/PentaC, PentaD	Binary: MSI-H (2 ≥ /5); MSS (< 2/5)	FFPE/1 ng DNA	R	[170]
PlentiPlex™ MSI (PentaBase)	RT-PCR, Capillary Electrophoresis/compatible instruments (e.g. BaseTyper™ 48.4 RT-PCR system)	Fragment Analysis Software	BAT-25, BAT-26, NR-21, NR-24, MONO-27/D17S250, D2S123, D5S346	Ternary: MSI-H (2 ≥ /5); MSI-L(1/5); MSS (0/5)	FFPE; FF/1–40 ng	R	[170, 171]
TrueMark™ MSI Assay (ThermoFisher)	RT-PCR, Capillary Electrophoresis/SeqStudio or ABI 3500 Series Genetic Analyzers	TrueMark™ MSI Analysis Software	BAT-25, NR-24, NR-21, BAT-40, CAT-25, NR-22, NR-27, ABI-19, ABI-20B, ABI-17, ABI-16, BAT-26, ABI-20A/TH01, PentaD	Binary: MSI-H vs MSS	FFPE/2 ng DNA	R	[170, 172]
ProDx® MSI Analysis System	FM-PCR, Capillary Electrophoresis/ABI 3500Dx Genetic Analyzer	Fragment Analysis Software	BAT-52, BAT-56, BAT-59, BAT-60, BAT-25, BAT-26, NR-21, MONO-27	Ternary: MSI-H (2 ≥ /5); MSI-L(1/5); MSS (0/5)	FFPE/5–10 ng DNA	R	[123]
Bio-Rad® ddPCR MSI Assay	Droplet Digital PCR/Bio-Rad QX200 Instrument System	QuantaSoft software v.1.7.4 (Bio-Rad)	BAT-25, BAT-26, NR-21, NR-24, MONO-27	Binary: MSI-H (2 ≥ 5); MSS (0 or 1/5)	FFPE; FF; plasma or serum samples/NA	NR	[89]
Idylla™ MSI Test (Biocartis)	RT-PCR, High-Resolution Melting Curve Analysis/Biocartis Idylla™ System	Idylla™ Explore	ACVR2A, BTBD7, DIDO1, MRE11, RYR3, SEC31A, SULF2	Binary: MSI-H (2 ≥ /7); MSS (< 2/7)	5 μm (50–600 mm^2^) or 10 μm (25–300 mm^2^) FFPE tissue section/no DNA extraction	NR	[173]
QiaXcel Advanced System/pilot phase	PCR/QiaXcel Advanced System	QiaXcel System	BAT-25, BAT-26, BAT-40/D2s123, D10s197, D13s153, D17s250, D18s58, D5s346, MycI	Binary: MSI-H vs MSS	FFPE/5 ng DNA	R	[174]
*HSP110* (T17) PCR	E-*ice*-COLD-PCR/LightCycler, ABI 3500Dx Genetic Analyzer	NA	T(17) in *HSP110* gene	Suitable for improving analytical sensitivity of MSI testing; not for MSI status classification	FFPE/20 ng DNA	NR	[175–177]
Drop-off ddPCR	ddPCR/Bio-Rad System	NA	BAT26, ACVR2A/DEFB105A/B	Bimodal distribution of MSI allelic frequencies	FFPE; plasma; serum/NA	NR	[144]
Titano MSI Test (Diatech Pharmacogenetics)	FM-PCR, Capillary Electrophoresis/compatible Genetic Analyzers	Fragment Analysis Software	BAT25, BAT26, D2S123, D17S250, D5S346, BAT40, D18S58, NR21, NR24, TGFβRII/TPOX, TH01	Binary: MSI-H vs MSS	FFPE; FF; WB (only CRC)/20 ng DNA	R	[178]
Agilent TapeStation 4200 platform	Capillary Electrophoresis/Bioanalyzer	TapeStation Analysis Software	BAT25, BAT26/D5S346, D2S123, D17S250	Binary: MSI-H vs MSS	FFPE/20 ng DNA	R	[178]
VarTracer® MSI qPCR Assay (NuProbe)	Multi-blocker displacement amplification (mBDA) & qPCR/BioRad CFX96	VarTrace MSI Software	BAT-25, BAT-26, NR-21, NR-24, MONO-27	Ternary: According to Ct value for each out of the marker: MSI-H (> 2/5); MSI-L(1/5); MSS (0/5)	FFPE; WB/1.5–15 ng DNA per reaction (3–30 ng per specimen); ideally 20 ng (10 ng DNA per tube)	NR	[179]

Explanatory notes: *Lab. equip.*, laboratory equipment; *FM-PCR*, fluorescent multiplex PCR; *ddPCR*, droplet digital PCR; *Ref.*, references; *NA*, not available; *R*, required; *NR*, not required; *MNRs*, mononucleotide repeats; *DNRs*, dinucleotide repeats; *MSI-H*, microsatellite instability - high; *MSI-L*, microsatellite instability - low; *MSS*, microsatellite stable; *FFPE*, formalin-fixed and paraffin-embedded; *FF*, fresh frozen; *WB*, whole blood

**Table 2:** List of microsatellite panels using tissue-based MPS strategies

Study	Panel/No. of tested loci	Seq. Platform	Paired samples	Tool	Tumour type	Analytical performance/reference methods
[180]	3 In-house panels/3 154, 230, 23	NextSeq 500	R	mSINGS, mSILICO	CRC	NA
[112]	TruSight™ Oncology 500/130 nc-MNRs	NextSeq™ 550Dx	NR	In-house	6 cancer types	98.0% (106) specificity/pentaplex MSI-PCR
[108]	OncoPanel AMC version 3/85 MNRs	MiSeq	NR	NA	CRC	92.1% sensitivity; 100.0% specificity for MSI-H/NA
[181]	5 MNRs; 2 DNRs	MiSeq	R	In-house	CRC	100.0% sensitivity; 100.0% specificity/pentaplex MSI-PCR or 9 loci NCI/Bethesda panel
[109]	40 unspecified loci	MiSeq	NR	In-house	7 cancer types	98.0% (98/100) concordance/pentaplex MSI-PCR
[14]	17 MNRs	MiSeq	NR	In-house	CRC	100.0% sensitivity; 100.0% specificity/pentaplex MSI-PCR
[81]	1 880 MNRs	In-house FoundationOne assay	NR	In-house	Large cohort	Combined concordance (con.) 97.0% (65/67)/pentaplex MSI-PCR and IHC; 95.0% con./MSI-PCR; 100.0% con./IHC; 97.0% con. with 95.0% sensitivity and 98.0% specificity to comparable methods [101]
[182]	BROCA/146 MNRs	HiSeq 2500	R	mSINGS	Prostate cancer	NA/pentaplex MSI-PCR
[107]	smMIPs panel/111 MNRs	NextSeq 500	NR	mSINGs	CRC, prostate, endometrial cancer	95.8–100.0% sensitivity; 100.0% specificity/pentaplex MSI-PCR
[106]	Caris MI TumourSeek/7 317	NextSeq	NR	In-house	26 cancer types	100.0% sensitivity; 99.9% specificity (CRC), 95.8% sensitivity; 99.4% specificity (all cancer types)/pentaplex MSI-PCR
[105]	UW-OncoPlex/15 MNRs	NA	NA	mSINGs	NA	100.0% sensitivity; 100.0% specificity/pentaplex MSI-PCR
[183]	Caris MI TumourSeek	NA	NA	In-house	12 cancer types	95.8% sensitivity; 99.4% specificity/pentaplex MSI-PCR
[103]	ColonCore/22 MNRs	NA	R	MSI-ColonCore	CRC	97.9% sensitivity (47/48); 100.0% specificity (37/37)/pentaplex MSI-PCR and IHC
[117]	NA	NA	R	MANTIS, MSIsensor, mSINGs	6 cancer types	97.2% sensitivity; 99.7% specificity for MANTIS, 96.5%; 98.7% for MSIsensor, 76.06%; 99.7% for mSINGs/NA
[76]	MSK-IMPACT/1000–1500 unspecified loci	Targeted NGS dataset	R	MSIsensor	66 cancer types	96.1% sensitivity; 98.5% specificity (MSS vs MSI-H); 100.0% sensitivity; 99.3% specificity (MSI-H in CRCs and uterine endometrioid cancers), 96.6% sensitivity; 100.0% specificity (MSI-H in other tumour types)/pentaplex MSI-PCR
[101]	NA	HiSeq 2500	NR	In-house	CRC	100.0% sensitivity; 99.0% specificity in detection of MSI-H/pentaplex MSI-PCR
[102]	17 MNRs	MiSeq	NR	mSINGs	78 cancer types	97.1% sensitivity (34/35) in MSI positive samples; 100.0% specificity (42/42) in MSI negative samples/pentaplex MSI-PCR

Explanatory notes: *Seq. Platform*, sequencing platform; *Ref*., references; *NA*, not available; *R*, required; *NR*, not required; *nc*, non-coding; *MNRs*, mononucleotide repeats; *DNRs*, dinucleotide repeats

Currently, the gold standard MSI-PCR assay designed by Promega (Madison, USA), MSI Analysis System, Version 1.2, uses a consensus set of five relatively long homopolymeric MS sites in the human genome (Table 1) [89, 97, 98]. The limitations of conventional tissue-based methods, analysing a limited number of loci, along with the prognostic and therapeutic intervention value of MSI status, urge the necessity of more precise and rapid detection of MSI for better survival outcomes in CRC. Exome and whole-genome analyses under The Cancer Genome Atlas (TCGA) project [99] have revealed the potential of MPS technologies in MSI detection, based on the sequencing of 224 paired tumour vs healthy samples focusing on homopolymers ranging from 6 up to 10 nucleotides. Genome sequencing is the current state-of-the-art technology to screen for MSI events. At present, there are numerous MPS-based MSI detection methods, some designed for paired healthy and tumour samples, and some more recent ones aiming to reveal MSI events using no control specimens (Table 2). Moreover, these strategies represent an appropriate alternative, especially in non-Lynch-associated tumours, where the driving mechanisms of MSI can be more complex and involve more genes and unusual pathways [100].

## Targeted sequencing

MPS has mostly replaced IHC and PCR approaches in MSI status detection, as it enables to analyse dMMR and MSI status simultaneously from tumour specimens [101]. Thus, various MPS-based approaches have begun to emerge, including MSIplus, designed to evaluate MSI status together with mutations in *KRAS*, *NRAS* and *BRAF* genes. This method does not require a matched normal sample and instead uses an amplicon sequencing strategy. Such an approach enables effective targeting and a high depth of coverage for the loci of interest [102]. The ColonCore panel (Burning Rock, Guangzhou, China) was designed to detect the MSI status together with mutations in 36 CRC-related genes (including *KRAS*, *NRAS*, *BRAF*, hereditary CRC genes and other genes related to carcinogenesis and tumour development). In combination with this sequencing panel, Zhu et al. developed an algorithm suitable for MSI detection, revealing the MSI status of a sample by determining the percentage of unstable loci, without a paired specimen [103]. They validated this approach with a gold standard MSI-PCR and IHC, reaching a high concordance rate. Compared to MSIsensor [104] and mSINGS [105], it achieved a better result and seems to represent a robust method based on read-count distribution [103].

So far, many methods have been developed for the detection of MSI from targeted MPS data and have demonstrated their reliability, sensitivity and efficiency across various cancers, even those not typically screened, without the need for matched normal tissue [76, 81, 106–110]. Apart from developing one of these methods, Trabucco et al. [81] analysed additional alterations that should be common for MSI-H and MSS tumours. They identified genes enriched in MSI-H tumours that fell into common pathways, such as the phosphatidylinositol 3-kinase (PI3K), NOTCH and WNT pathways. Janus kinase/signal transducers and activators of transcription (JAK-STAT) and Hedgehog pathways were also found to be enriched, but only when regions of polymerase slippage were included. On the other hand, MSS tumours were consistently enriched in alterations of genes involved in the cell cycle across all tumour types [81]. Currently, TruSightTM Oncology 500 (TSO500), the innovative and accurate tumour-only pan-cancer workflow proposed by Illumina, is widely used for MSI screening. It may be regarded as a straightforward and cost-effective assay, allowing the detection of various DNA variant types [111, 112].

## Whole-exome sequencing

Many known MSI characteristics were identified using conventional strategies. However, these observations came from analysing only a few dozen loci in cohorts lacking sufficient size. A robust strategy using tumour exomes from TCGA Research Network to predict and examine MSI status across 18 cancer types was introduced [68]. It worked with 2.7% of all MSs identified through the human genome (more than 500,000 loci), representing 95.9% of all coding MSs and 98.4% of MSs located in splice sites. Based on this approach, MOSAIC, a classifier for predicting MSI status, was introduced (see section “Bioinformatics: essential element for MPS-based MSI assessment”). It reclassified MSI-L tumours as MSS, supporting previous assumptions that MSI-L is not a distinct class [43, 113]. Another tool, MSIpred, was developed and trained on MSS (encompassing MSI-L) and MSI-H tumours. This tool also seems to be reliable for MSI classification and capable of distinguishing between different tumour types as MSS and MSI-H tumours possess distinct somatic mutational loads [114].

Since whole-exome sequencing (WES) is becoming increasingly used in clinical care and 3PM, there is a growing need to use this data for MSI status detection. Some of the emerging methods are turning out as valid, adequate and sensitive [115]. New computational tools have been developed to extract MSI status from whole-exome data, but each method, process, tool and even the whole pipeline should be validated before it is integrated into routine usage [116]. Accordingly, MANTIS, one of the tools developed for the above purpose [117], was compared with mSINGS [105] and MSIsensor [104], and their performance and accuracy were validated. MANTIS displayed high accuracy and robustness across many cancer types and loci. Despite some discrepancies [68, 102, 114], the above tools may reliably complement the conventional MSI-PCR method, and may soon become stand-alone gold standards for MSI status identification.

## Whole-genome sequencing: a tool for MSI-related cancer detection

Genome-wide genotyping of STRs offers extensive potential to transform applications in cancer research and 3PM beyond the study of conventional MSI. In whole-genome sequencing (WGS), STRs have presented a broad range of challenges as diagnostic markers. The screening of MSI using a large-scale WGS approach may yield a continuously valued MSI score that may have greater utility in determining genomic MSI events [117]. One of the common issues for robust MSI detection on the whole-genome scale is the usage of sequencing data obtained from low-coverage analyses [76]. However, the performance of the software for the assessment of MSI from WGS data is severely affected by the depth of sequencing. Thus, determining the minimum sequencing depth of coverage for making MSI calls and characterising the loss of detection power in low-coverage regions seems helpful [118]. A genome-wide survey of MSI presented by Cortes-Ciriano et al. revealed that MSI events may affect as many as 300,000 loci. Non-coding MSI events occurring in regulatory elements may function as cancer drivers [44]. Although there are numerous computational tools for screening of somatic MSI, only few use low-coverage WGS data. MSIClass, developed by Maruvka et al. [119] using ultra-low coverage data (as low as 0.05 ×), could accurately detect somatic MSI events.

As the era of MPS began, this method was also recognised as useful in the field of cancer assessment, especially for detecting MSI status in tumours characterised by low MSI frequency, since MSI testing is not performed systematically. It has been shown that each cancer type may have its own MSI signature, so an important benefit of MPS is the ability to include numerous MS loci. The number and selection of the analysed loci may affect the accuracy of the MSI calling method. It may therefore be helpful to find an optimal number of loci for analysis with each tool to ensure its optimal performance [117]. There are many MSs found across the human genome [44, 68], and their behaviour may vary across different malignancies. The cancer-specific MSI landscape promises potential predictive power with huge implications for clinical diagnosis but it may only be uncovered by WES or WGS. Most MSs are located in non-coding regions of the genome and may affect gene regulation, consequently leading to carcinogenesis.

## Technical and biological limitations of MPS-based MSI detection approaches

MSI detection through MPS is a very complex strategy involving many obstacles and challenges. Technical issues in MSI calling involve uneven genome coverage and artefacts in sequencing technology. It has been known for a long time that the frequency of errors (in both PCR or sequencing) tends to increase with homopolymer length [120]. Among all available sequencing platforms, Illumina has been shown to be the most suitable for these stretches due to its low error rate within monomeric tracts. The average error rate has been shown to range from 0.002% for homopolymers of 2 bp in length to 2% for 17-bp regions [121]. All in all, using homopolymeric tracts as diagnostic markers in MSI screening shows a significantly higher sensitivity in MSI-positive cancers in contrast to polymorphic dinucleotide repeat markers. In general, mononucleotide markers are preferred over higher repeat stretches. It seems a bit confusing, as multiple studies have shown that dinucleotide repeats are better for detecting MSI-L cancers, most of which are MMR-proficient, while mononucleotide MSI markers are far more effective in detecting dMMR tumours [122].

A major challenge in most strategies based on the analysis of homopolymeric markers is the detection of small indels [118]. Some evidence has suggested the number of repetitions under seven as a poor indicator; thus, longer mononucleotide repeats (> 15 bp) have been preferred [58]. Such repeats, however, are more prone to errors due to PCR or sequencing. Therefore, short repeats seem to offer an alternative, as they are also more monomorphic than longer variants, not requiring analysis of matched normal tissue [14]. There are hypotheses that longer homopolymeric repetitions could easily increase the sensitivity of the monomeric tract, thus improving the performance of MSI screening. A study by Wu et al. [123] performed on CRC patients demonstrated that the ProDx® MSI system containing four additional markers (BAT-52, BAT-56, BAT-59 and BAT-60) (Table 1) brought a minor improvement in detection sensitivity, similar to the prior finding of enhanced detection sensitivity by long mononucleotide repeat markers in colon polyps. Despite the above, it may be argued that long homopolymers might be ultimately better than shorter ones as more extensive changes are much more reliable when calculating MSI scores [123].

Detecting MSI events using not only homopolymer markers may also be difficult due to technical artefacts associated with FFPE specimens, routinely used in translational research settings [124]. The preparation of FFPE blocks may result in remarkable changes in DNA structure, such as deamination of C to T or transition of G to A, which may be partially resolved by enzymatic treatment with uracil DNA glycosylase and/or 5-methylcytosine DNA glycosylase, thus reducing background noise and false positive results [125].

Several factors pose a computational challenge for MSI detection by MPS, such as the errors induced during the sequencing-by-synthesis, the difficulties of alignment of repetitive DNA motifs due to the short read length and the low accuracy of indel calling [126]. Further improvements are expected to help reduce the high risk of interpretation errors and the chance of incorrect indication of patients for immunotherapy. Reliable algorithms successfully addressing this problem will likely take longer to develop.

Targeted sequencing allows efficient testing for MSI while screening for additional relevant genomic information. Moreover, MSI status may be reliably determined regardless of the specific baits on the MPS panel, as long as there is sufficient genomic coverage. Validation studies have shown the method to be sensitive and specific, with high concordance to traditional methods, enabling confident assessment of MSI status without the requirement for matched normal tissue [105]. However, dependence on read coverage represents the main limitation of MPS-based MSI detection compared to MSI-PCR and IHC [117]. We should also keep in mind that, similarly to PCR, MPS reveals the effects of dMMR without revealing the underlying cause.

## Bioinformatics as an essential element for MPS-based MSI assessment

**Fig. 1:**
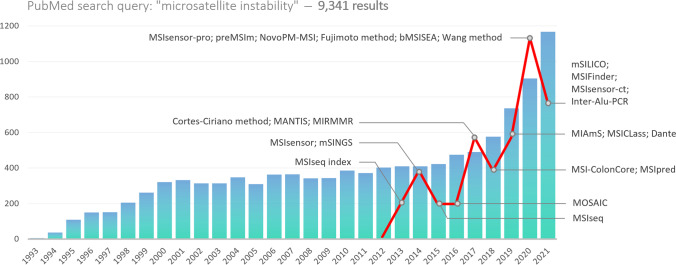
Annual distribution of PubMed search results for the term “microsatellite instability” as of December 21, 2022. The red curve represents the number of emerging bioinformatics tools for MPS-based MSI analysis. There seems to be a correlation between the increasing number of publications and the emergence of novel tools

In terms of 3PM objectives, bioinformatics offers improved diagnosis at the genomic level with earlier detection and more targeted therapy for effective personal disease monitoring [127]. Computational tools needed for MSI analysis may be easily integrated into the existing pipelines commonly used to detect other mutations (e.g. SNPs, CNVs) from sequenced genomic data. A retrospective analysis of previously generated MPS data is also possible. Various tools for detecting MSI status from MPS data are available (Fig. 1): some assess MS loci directly in DNA, while others indirectly, e.g. through the analysis of somatic mutations [128, 129]. There are other tools designed to determine MSI status from RNA-seq data (Table 4) [130]. Since these tools do not use the same sequencing data for input, it is not possible to compare their performance.

In the context of MPS-based algorithms usually focused on comparing the repeat lengths of several homopolymers, the majority of MSI detection tools are intended for a limited number of cancer types. There are two main types of MSI analysis. The mutation burden approach is limited by the need for large and costly sequencing panels, ranging from targeted to WGS panels, as the tumour mutation burden (TMB) calculated from small panels may deviate from reality. The second limitation is that cut-off values between MSS, MSI-L and MSI-H have to be defined for every sequencing panel. The second type of analysis is the read-count distribution approach [103], which is more compatible with smaller and cheaper sequencing panels compared to the mutation burden approach. With regard to cut-off values, this approach could be more versatile because cut-offs can be defined for any given panel using the percentage of unstable loci.

Several issues must be considered when selecting an appropriate tool for MSI analysis (Table 4). First, the type of biological material for nucleic acid extraction. Since tissue samples were commonly used for MSI detection, most tools were designed with them in mind. However, cell-free nucleic acids from liquid biopsy are steadily gaining ground and tissue-based approaches may not be compatible, as sensitivity and other metrics differ between these types of samples. The second important issue is related to the sequencing approach used for data generation. With the decreasing cost of DNA sequencing [131], targeted or panel sequencing, WES and even WGS strategies are becoming available. They differ in the range of the analysed regions and consequently in the amount of generated data that need to be processed in the downstream analysis. Some of the available tools can adequately work with all types of such data (MSIsensor-pro, MSIseq, Cortes-Ciriano method, mSING), while others are validated only on specific datasets (MSIsensor, MOSAIC, MANTIS, MSI-ColonCore, mSILICO, MIMcall, etc.) (see Table 4). Such tools may not process other datasets correctly, or data size itself may represent a limitation if the given tool has been designed for smaller panels. Many tools detect MSI, but are also capable of detecting TMB and may be also applied to predict the ICI efficacy [64–66].

Despite all the talk about decreasing costs, larger applications such as WGS are still too expensive for most labs worldwide. Low-coverage sequencing is a cheaper solution; however, to our knowledge, not many tools support the analysis of low-coverage data [119]. Technical differences between tools should be considered, including the programming language, availability, interface (command line and/or graphical user interface), up-to-date service and support. The choice of the right tool should be carefully considered based on the input data and the requirements of the downstream analysis. Ongoing development will lead to improved performance, thus offering new tools to enhance early prevention, diagnosis, prognosis and patient stratification regarding proper anti-cancer treatment and follow-up.

## Liquid biopsy: an instrument of MSI-related cancer screening

Multiple tissue-based tests are available for MSI status assessment, mainly using IHC and PCR [3, 19–22]. Although they are regarded as a gold standard, they come with inherent limitations, mainly due to the invasive nature of tissue biopsy. Disadvantages include the difficulty or impossibility of repeated sampling, the requirement of an adequate amount of tumour tissue with a high concentration of cancer cells and the routine need for paired control healthy tissue that is unavailable or infeasible to obtain in some patients. Secondly, tumours typically display genetic heterogeneity, so test results may be ambiguous due to the heterogeneous expression of MMR proteins, and tissue biopsy may not fully capture tumour diversity. Moreover, test performance often requires specialised equipment, but also an expert pathologist who is needed to interpret the results (in the case of IHC), which, in turn, may be a subjective and error-prone process [132–134]. Thirdly, there is the possibility of false positive or false negative results due to artificial loss of expression, missense mutations in MMR genes or polymorphisms in MSs, which may be termed as technical limitations [100, 135]. Traditional sampling may not be applicable in patients with inadequate tumour tissue or tumour localisation with limited accessibility [66, 136].

**Fig. 2:**
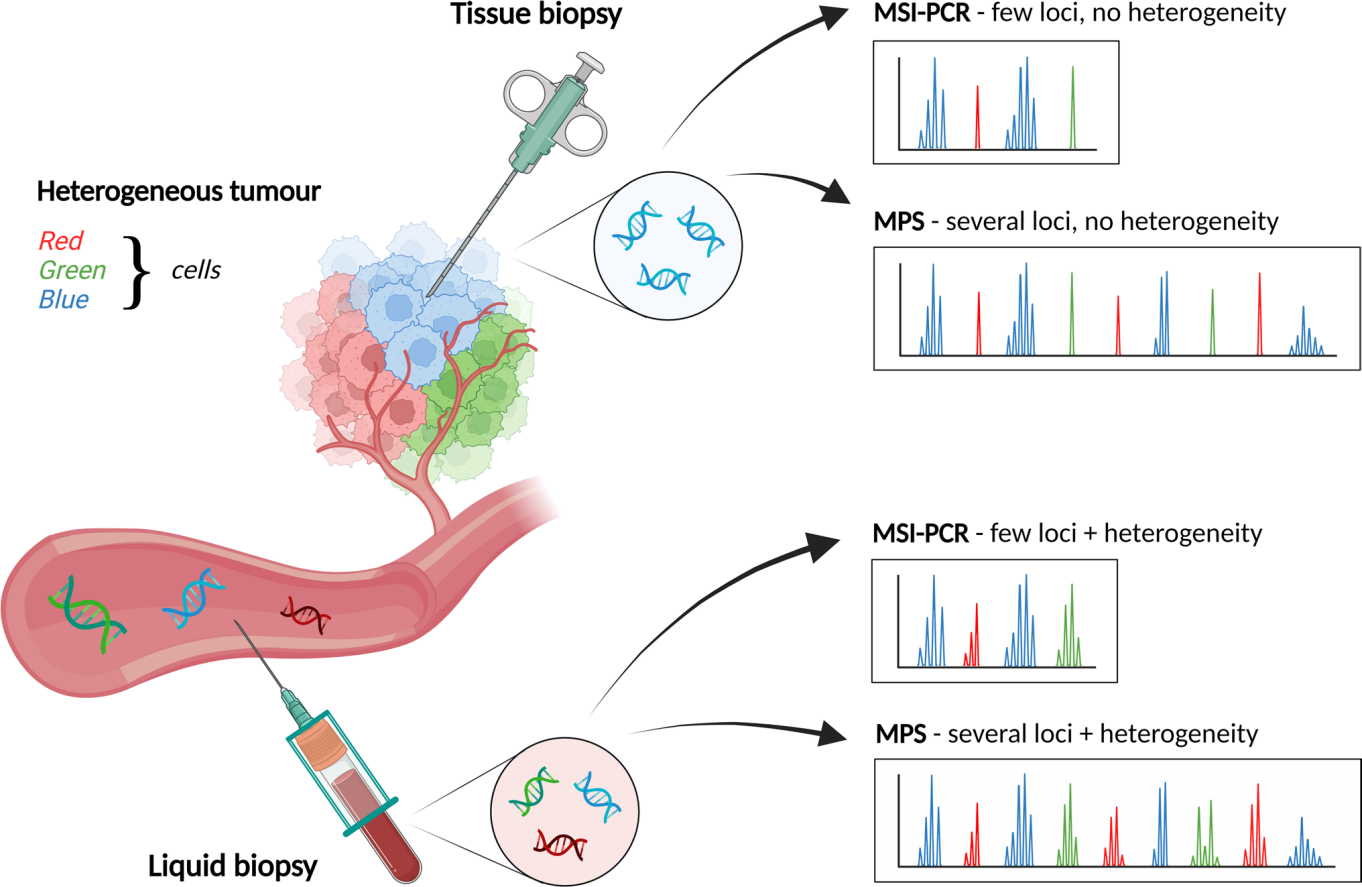
Since tissue biopsy analyses only a selected portion of the tumour, it may not capture its overall heterogeneity (*only blue cells*). As DNA from the entire tumour enters the bloodstream, liquid biopsy is able to capture ctDNA from all populations of tumour cells (*red*, *green* and *blue cells*). Given that MPS strategies can capture several MS markers in a single assay, these methods, combined with liquid biopsy, are the most representative approach for the personalisation of MSI detection (Created with BioRender.com)

Therefore, an increasing number of clinical laboratories are seeking to implement circulating tumour DNA (ctDNA) sequencing into their routine to provide 3PM care [137, 138]. It is expected that liquid biopsy-based methods will develop further in the near future and will be widely applied in the detection of early cancer and other diseases, disease monitoring and personalised therapy [139]. Thus, using liquid biopsy in combination with MPS in MSI assessment is an attractive and promising approach with many advantages in the field of 3PM. Contrary to conventional tissue biopsy, the main benefit of liquid biopsy in MSI testing is the minimally invasive procedure without any pre-requirement and with the possibility of repeated sampling, which is essential to monitor disease progression, treatment response or resistance to therapy. ctDNA comprises genomic changes that are hallmarks of cancer and thus may be used as a potential surrogate for the entire tumour, allowing diagnosticians to deal with its genetic heterogeneity (Fig. 2) and capture the mutational landscape of the entire tumour or the TMB [140–142]. This approach seems to yield a high concordance rate with tissue biopsy-based detection and high specificity, precision and sensitivity, as reported by several authors [126, 143–145]. Liquid biopsy-based methods have the potential to enhance the utility of tumour detection assays to help clinicians decide on immunotherapeutic treatment [143, 146].

Although liquid biopsy offers great potential in patient management [147–149], the assessment of MSI through ctDNA or cell-free DNA (cfDNA) is still in development due to various limitations. Several challenges need to be addressed before it is implemented into routine clinical practice and personalised medical service. One of the challenges is related to liquid biopsy in general because there is a lack of standard procedures, protocols and guidelines for sample collection, preparation, processing and storage, as well as for validation of studies. This could significantly affect the analytical stage and lead to difficulty in comparing results across different approaches [150–152]. Although liquid biopsy seems to be a sensitive procedure with high precision and accuracy [126, 143–145, 153], there is limited evidence regarding its sensitivity and accuracy in a diagnostic setting [154]. Next, there are cases with a low tumour fraction in the circulation (low concentration of cfDNA, ctDNA or CTCs). Artefacts or technical noise may also occur due to polymerase slippage resulting in false positive or false negative findings, while low signal-to-noise ratio may indicate contamination by non-tumour cells [151, 153, 155–157]. The final challenge to be addressed is related to the PCR- or MPS-based methodology used for analysis (see the previous chapters).

To summarise, detection of MSI status through liquid biopsy using MPS seems to be a promising approach, not only in early detection, predictive diagnostic and targeted prevention, but also in therapy management and consecutive monitoring of treatment response. Such approaches also offer diagnostic possibilities to patients at risk of developing MSI-associated disease even without a family history or to patients whose tumour tissue is unavailable, difficult to obtain or its acquisition is contraindicated. However, due to various technological and bioinformatic challenges, MSI detection through liquid biopsy using MPS is still in development, and further progress is needed before implementation into routine clinical practice and personalised medical services.

## Current methods based on liquid biopsy for MSI status detection

To date, there is limited knowledge about the utility of liquid biopsy in determining the MSI status, but some approaches and methods are emerging (Table 3), mainly thanks to the significant progress made towards improving the resolution of existing tissue-based approaches and adapting them to liquid biopsy. In recent years, many groups attempted to validate MPS-based approaches using plasma ctDNA for MSI detection with promising results [158, 159]. Using a ctDNA-based approach will also allow increased access to checkpoint inhibitors in a pan-tumour setting. This would be relevant in targeted prevention of cancers where routine MSI testing is contraindicated or when tissue is not available or accessible [159].

**Table 3:** List of blood panel-based MPS strategies

Study	Panel/method	Sequencing platform	No. of tested loci	cfDNA input	Analytical performance/reference methods
[143]	Guardant360®CDx	NextSeq500/550 or HiSeq 2500	90 MS loci (MNRs, DNRs)	5–30 ng	LOD: 0.09–0.1% ctDNA content 86.6% sensitivity; 99.5% specificity/pentaplex MSI-PCR, IHC, MSI-NGS on tissues
[161]	FoundationOne® Liquid CDx	In-house MPS-based device	~ 2000 MS (MNRs, DNRs, TNRs)	20–30 ng	LOD: 0.8% unstable loci/NA
[184]	NA	MiSeq	24 MNRs	100 ng	98.0–100.0% sensitivity; 98.0–100.0% specificity/tissue MSI, MMR genes sequencing
[160, 161]	OncoLBX	MiSeq	5 MNRs	20–30 ng	LOD: 2.0% tumour fraction/NA
[146]	In-house	HiSeq 2000/2500	5 MNRs	5–250 ng	78.3% sensitivity (18/23); 100.0% specificity (6/6)/pentaplex MSI-PCR, IHC, MSI-NGS on tissues

Explanatory notes: *NA*, not available; *MNRs*, mononucleotide repeats; *DNRs*, dinucleotide repeats; *TNRs*, trinucleotide repeats; *LOD*, limit of detection; *ctDNA*, circulating tumour DNA; *MMR*, mismatch repair; *IHC*, immunohistochemistry

The potential of cfDNA in cancer diagnosis leads to the development of various tests, such as the OncoLBx test designed as a combination of a single-molecule sequencing (SMSEQ) platform with a pan-cancer MPS panel targeting 75 genes and five homopolymer marker sites. During its validation, OncoLBx demonstrated that it could detect variant allele frequencies of ≥ 0.1% for SNVs and indels, ≥ 0.5% for fusions, ≥ 4.5% copies for CNVs and ≥ 2% for MSI, displaying a specificity of ≥ 99.999% for all variant types [160]. Willis et al. demonstrated robust analytic performance for MSI detection on the Guardant360® CDx test (Guardant Health, Redwood City, CA, USA), an FDA-approved liquid biopsy, which had been previously validated for the detection of four other variant types. This approach proved to be highly sensitive compared to tissue-based methods while maintaining very high specificity [143]. To our knowledge, it is the first ctDNA-based landscape analysis of MSI in a large advanced pan-cancer cohort. Georgiadis et al. developed a protocol for simultaneous MSI and TMB detection using a non-invasive method which is also effective in predicting response to immune checkpoint therapy. They achieved 100% sensitivity and specificity during the evaluation of their matched tumour and normal tissue samples [146].

A few months later, a different approach utilising a quantitative drop-off ddPCR was introduced (see Table 1). This method is able to target long and short repetitive genome regions, so it may be applied in MSI status determination and accurate ctDNA quantification. Although it is a 3-marker MSI-ddPCR assay, results suggest that it is possible to develop assays targeting additional markers identified by cancer exome sequencing for improving the clinical sensitivity of MSI testing across different cancer types. The method can also be applied to screen formalin-fixed and paraffin-embedded (FFPE) tumour samples and does not require paired tumour-normal samples for reliable MSI identification [144]. As it aims for absolute quantification of ctDNA or MSI sequences, it has the potential to monitor ctDNA in patients longitudinally and may significantly improve 3PM strategies. Apart from the above-mentioned Guardant360® CDx, there is another FDA-approved liquid biopsy test, for tumour profiling, including MSI detection, FoundationOne® Liquid CDx (Foundation Medicine, Cambridge, MA, USA). In a validation study, the proposed test achieved high sensitivity and specificity and proved to be sufficiently robust and comparable to other MPS-based broad molecular profiling liquid biopsy assays [161].

The strategies applied by Guardant360® CDx, FoundationOne® Liquid CDx, OncoLBx and Georgiadis et al. (Table 3) all employ hybrid-capture enrichment of target regions and use molecular barcoding to avoid false positive events arising due to technical PCR errors. The error correction process they use reduces background noise and enables variant calling with high sensitivity, specificity and accuracy. Along with blood MSI status, many of these approaches offer detection of other variant types, including the determination of blood-based tumour mutation burden (bTMB), a complementary predictive biomarker that may inform ICI treatment.

However, detection of MSI events from blood adds another specific challenge. Since the highly fragmented nature of ctDNA and the low ctDNA fraction in body fluids (as low as 0.01% in early stages of cancer) along with technical artefacts generated during library preparation, amplification or sequencing lessen the analytical sensitivity, determination of MSI from plasma samples requires methods that are, above all, highly sensitive [153]. The sensitivity of 98% and specificity of 100% in deep sequencing of samples with a minimum of 1% ctDNA by the ColonCore panel were observed using bMSISEA (blood MSI signature enrichment analysis) detection algorithm. This tool also showed high sensitivity of 94.1% and specificity of 100% with a minimum ctDNA fraction at 0.4% (Table 4). Moreover, it efficiently distinguished between MSI-H and MSS samples [162]. MSIsensor-ct [163] deployed on the NovaSeq platform (Illumina) using cfDNA sequencing data yielded 100% sensitivity and 100% specificity on 39 plasma specimens. The minimal ctDNA fraction for reliable assessment of the MSI status should be at least 0.05% with a sequencing depth over 3000 × .

**Table 4:** List of computational tools

Sample	Tool	Strategy	Prog. Language	Description	Ref.
Tissue	Cortes-Ciriano method	WGS, WES	NA	The tool allows assessing the difference in reading length distribution at each locus using the statistical Kolmogorov–Smirnov test	[44]
	MSIsensor	WES	C + + , R	This software tool is suitable for deriving MSI status in standard paired tumour/normal sequencing data and automatically detecting somatic changes at MNRs. It computes length distributions of MS per site and subsequently uses these to compare observed distributions in both samples statistically	[104]
	MSIsensor-pro	WGS, WES, TGS	C + +	MSIsensor-pro is an updated freely available version of the MSIsensor tool that quantifies polymerase slippages for each tumour sample with no need for control	[185]
	MSIseq	WGS, WES, TGS	R	MSIseq is an R statistical language package for classifying MSI status based on four machine-learning frameworks operating on MPS data	[128]
	MSIseq index	RNAseq	NA	MSI prediction method utilises RNA sequencing data comparing indels in defined MSs vs all MSs	[130]
	MOSAIC	WES	Python, Perl, R	MOSAIC is a command-line interface tool whose algorithm is based on the average gain in the number of MS alleles and locus instability. It corrects for class imbalance in its cross-validation training procedure (with an approximately 3:1 MSS-to-MSI-H ratio), making predictions in new cancer types without any prior assumption about the expected prevalence of MSI-H tumours	[68]
	mSINGs	WES, TGS	Python	mSINGs is an MSI-detection tool based on the detection of altered read count distribution which evaluates each MS locus within a homopolymer marker set and reports MSI status by specifying certain cut-offs	[105]
	MANTIS	WES	Python	This tool could be incorporated into the existing pipelines, applicable to a variety of tumours with varying numbers of MNRs. It analyses the instability of paired samples by aggregating loci instead of individual locus differences to evaluate general instability present in a tumour sample since the data from the paired sample acts as an error-correcting baseline	[117]
	MSI-ColonCore	TGS	NA	This tool predicts the MSI status according to Z-score, sorting samples into MSI-H, MSI-L or MSS groups	[103]
	mSILICO	TGS	NA	mSILICO is a TGS-based computational method using a custom marker panel to assess MSI	[180]
	NA	WGS	Perl	Fujimoto method implements MIMcall (somatic indel caller) in calculating microsatellite error rates for the various MS motifs on the X chromosome. A sample is considered to be MSI if selected microsatellites show instability in ≥ 3% loci	[55]
	MIAmS	TGS	NA	MIAms (Microsatellites Instability by AMplicon Sequencing) is a command-line tool for routinely predicting MSI status without analysing normal tissue samples	[186]
	MSIpred	WES	Python	Microsatellite Instability Predictor, implementing SVM classifiers, is a robust tool for pan-tumour MSI classification, freely available as a Python 2 package	[114]
	preMSIm	TGS	R	preMSIm (Predicting MSI from mRNA) is a publicly available tool for predicting MSI from the expression profiling of a 15-gene panel	[187]
	NovoPM-MSI	TGS	NA	NovoPM-MSI is an algorithm that uses a target panel consisting of a set of enriched MNRs. MSI score is reported as a fraction of unstable loci by comparing length distribution in paired samples	[110]
	MSIFinder	TGS	Python	MSIFinder, a python package for automatic MSI classification that uses a random forest classifier (RFC)-based genome sequencing, a machine learning technology. The length of analysed MNRs ranged from 10 to 34 bp	[188]
	MIRMMR	WGS	R	MIRMMR predicts MSI status using methylation and mutation information and highlights genetic alterations significantly contributing to MSI screening	[189]
	Dante	WGS, WES, TGS	Python	A tool for analysing STRs from MPS data, originally designed for TGS data. It does not require the mapping of reads to a reference genome and is designed on the specific sequencing characteristics of STR alleles	[92]
Blood	bMSISEA	TGS/ColonCore panel	R	The blood MSI signature enrichment analysis is a method for blood-based MSI screening/100.0% (27/27) specificity; 94.1% sensitivity (16/17) for ctDNA content > 0.4%; 98.8% concordance between bMSISEA and IHC	[162]
	MSIsensor-ct	TGS	NA	A tool based on machine learning, dedicated to detecting MSI status using cfDNA sequencing data with a potential stable MSIscore threshold of 20%, with no requirement for pre-constructed baseline control/100.0% sensitivity; 100.0% specificity; LOD: 0.05% ctDNA with a minimum coverage of 3000 ×	[163]
	Wang method	WES	NA	This method enables detection of the difference in reading length distribution at each locus. The proportion of unstable loci among the selected 100 loci is used to calculate the blood-based MSI score/82.5% sensitivity (33/40); 92.6% specificity (201/209); 94.0% overall concordance (234/249); LOD: 0.5% ctDNA at an input of 30 ng DNA	[190]
	MSICLass	WGS	NA	A tool for MSI status classification from shallow WGS data which automatically detects somatic MSI events. The sequencing coverage as low as 0.05 × is sufficient for accurately calling the MSI status	[191]
	Inter-Alu-PCR	TGS	NA	This tool combines MNR enrichment and a custom MSI-tracer algorithm to compare the MNR’s read length. It can distinguish the cfDNA from cancer patients with MSI and samples from healthy individuals	[192]

Explanatory notes: *Prog. Language*, programming language; *Ref.*, references; *NA*, not available; *TGS*, targeted sequencing; *WES*, whole exome sequencing; *WGS*, whole genome sequencing; *MSI-H*, microsatellite instability-high; *MSI-L*, microsatellite instability-low; *MSS*, microsatellite stable; *cfDNA*, cell-free DNA; *ctDNA*, circulating tumour DNA; *STRs*, short tandem repeats; *MNRs*, mononucleotide repeats; *LOD*, limit of detection; *IHC*, immunohistochemistry

## Conclusions and outlook in the framework of 3PM

End-of-life care for advanced cancer patients is generally considered of poor quality [164]. Moreover, huge expenditures are required during the patient’s last year of life to increase the quality of palliative care. Since generalised approaches in cancer management come with an enormous socio-economic burden, personalised medicine is a vital aspect of palliative care. Various 3PM concepts are designed to develop maximum efforts to improve individual outcomes at the initial care level [165]. Implementing such a strategy is crucial to save lives while ensuring cost efficacy of medical services provided to the population [166]. Innovations in less invasive diagnosis and high-throughput technologies are accelerating, promoting and revolutionising a shift from decades-old testing strategies to reactive 3PM approaches [167, 168]. Since the role of MSI status as a predictive biomarker for cancer immunotherapy had already been clarified, the era of personalised medicine has dawned in regard to screening MSI across different levels of the human genome, from individual marker sites (numbers ranging from several to hundreds of sites) to comprehensive large-scale pan-cancer analyses comprising thousands of evaluated loci.

MSI status as a promising biomarker with strong clinical significance used in the early diagnosis of LS and CRC patients positively correlates with survival outcomes and enables predicting the efficacy of immune checkpoint blockade therapy in solid tumours. This might improve individually tailored treatment decisions and follow-up strategies in MSI tumours. We hypothesise that comprehensive panels could now advocate universal MSI screening for all relevant individuals to provide better cancer prevention opportunities for them and their relatives. More effective treatment and surveillance for these individuals will result in increased survival and reduced costs of medical care. In addition, for the LS detection routine, MSI testing should be used as a universal MPS-based tumour screening approach. It turns out that most experts endorse the possibility of using it and recommend it as the first step in diagnosing LS and other diseases. Therefore, 3PM-based diagnostic approach using MPS technology is expected to significantly revolutionise the screening approach of this cancer-prone syndrome. Accurate diagnosis, precise management and accurate prevention are what the patient population urgently needs.

As their implementation into clinical practice is already in progress, 3PM-based approaches are becoming increasingly accessible to patients. However, to date, only a small number of them are applicable for the detection of MSI events in plasma, blood or tumour tissue with a low ctDNA fraction. As discussed in this review, technical and biological difficulties slowed down the introduction of these methods to clinical diagnostic laboratories. Therefore, newly available bioinformatics and computational algorithms must meet specific criteria, such as the ability to deal with unique characteristics of MS motifs, DNA slippage events or PCR errors introduced into homopolymeric sites during sequencing as well as alignment errors due to short read length.

We compiled a comprehensive list of high-throughput strategies and computational tools for MSI evaluation based on tissue and liquid biopsy. In the context of less invasive methods so far not used to the extent of their potential, there is growing evidence that ctDNA provides a highly concordant source of MSI-affected alleles. Liquid biopsy-derived methods thus increase patient access to standard-of-care-targeted therapies by quickly identifying the cases that are otherwise difficult to find due to tissue sampling limitations. With an increasing number of studies promoting the use of ctDNA and/or low-coverage sequencing data, many new in-house MSI screening tools are likely to be designed soon. In addition, using the MPS data enables analysis of other cancer-related signatures, facilitating the adoption of MSI clinical testing and supporting personalised cancer therapy. When the third generation of sequencing gains a foothold in cancer research, it will bring along its own tools for data analysis. Moreover, in the age of individualised and precise treatment, these strategies can no longer be ignored.

## Data Availability

Not applicable

## Abbreviations

3PM/PPPM: Predictive, Preventive and Personalised Medicine
MSI: Microsatellite instability
MSI-H: Microsatellite instability-high
MSI-L: Microsatellite instability-low
MSS: Microsatellite stable
TR: Tandem repeat
MS: Microsatellite
STR: Short tandem repeat
MMR: Mismatch repair system
dMMR: Mismatch repair deficiency
MPS: Massively parallel sequencing
CRC: Colorectal cancer
EC: Endometrial cancer
LS: Lynch syndrome
IHC: Immunohistochemistry
FFPE: Formalin-fixed and paraffin-embedded
TMB: Tumour mutation burden
bTMT: Blood-based tumour mutation burden
ICI: Immune checkpoint inhibitor
WES: Whole-exome sequencing
WGS: Whole-genome sequencing
SNV: Single nucleotide variant
CNV: Copy number variant
ctDNA: Circulating tumour DNA
cfDNA: Cell-free DNA
